# A new macrofossil ephedroid plant with unusual bract morphology from the Lower Cretaceous Jiufotang Formation of northeastern China

**DOI:** 10.1186/s12862-019-1569-y

**Published:** 2020-02-04

**Authors:** Yong Yang, Yingwei Wang, David Kay Ferguson

**Affiliations:** 1 State Key Laboratory of Systematic and Evolutionary Botany, Institute of Botany, Chinese Academy of Sciences, Beijing, 100093 China; 2 Beijing Botanical Garden, Institute of Botany, Chinese Academy of Sciences, Beijing, 100093 China; 3 Department of Paleontology, University of Vienna, 1090 Vienna, Austria

**Keywords:** China, Diversity, Ecology, Evolution, Ephedraceae, Gnetophytes, Jiufotang Formation, Lower Cretaceous

## Abstract

**Background:**

The evolution of the Jehol Biota of western Liaoning in China includes three phases, initiation in the Dabeigou phase, radiation in the Yixian phase, and decline in the Jiufotang phase. Numerous ephedroid macrofossils were reported from the Lower Cretaceous Yixian Formation. However, so far none has been found in the younger Jiufotang Formation (ca. 120.3 Ma) of western Liaoning.

**Results:**

Here we report a new species *Jianchangia verticillata* gen. et sp. nov. with unusual morphology from the Lower Cretaceous of the Jiufotang Formation, Lamadong Village, Jianchang County, Liaoning. This species is the first record of gnetophytes from the Jiufotang Formation. It is similar to other ephedroid species from the Yixian Formation in possessing linear leaves with parallel veins, jointed shoots with swollen nodes and longitudinally furrowed internodes, and ovulate cones possessing two whorls of bracts enclosing two chlamydosperms, but differs from all known species by the ovulate cone having multiple fine linear verticillate bracts.

**Conclusions:**

This study expands our knowledge about the diversity of early gnetophytes in the Lower Cretaceous, and demonstrates the lineage continuity of gnetophytes from the Yixian Formation to the younger Jiufotang Formation.

## Background

Modern gnetophytes consist of three monogeneric families: Ephedraceae (*Ephedra* L., 60 spp.), Gnetaceae (*Gnetum* L., 44 spp.), and Welwitschiaceae (*Welwitschia* Hook. f., 1 sp.) [1]. The three living families possess divergent characters: Ephedraceae are usually dichasially branched shrubs, rarely small trees or lianas, possess linear parallel-veined leaves at the swollen nodes, compact ovulate cones with only one distal pair/whorl of fertile bracts, and are distributed in cold and arid places; Gnetaceae are commonly lianas, rarely small trees, possess pinnately veined broad leaves at the swollen nodes, and ovulate spikes with multiple loosely arranged fertile bract collars, and occur in tropical/subtropical evergreen forests; while Welwitschiaceae are short and unbranched plants, have ovulate cones with many pairs of fertile bracts, have two giant persistent leaves with multiple parallel veins connected by cross-veins, and are restricted to arid coastal areas of southwestern Africa [1–9].

Despite their divergence, these plants consistently bear a unique chlamydosperm, i.e. one or two outer envelopes enclosing an inner ovule with the integument elongated beyond the envelope into a micropylar tube [10–16]. This unusual structure is different from the naked ovule in cycads, *Ginkgo* L., conifers, and from the angiospermous ovule completely enclosed within a closed carpel, thus displaying a seemingly transitional morphology between angiosperms and other groups of gymnosperms. Recent phylogenetic/genomic/phylotranscriptomic studies have suggested that the gnetophytes are sister to the Pinaceae, i.e. the gnepine hypothesis [17–20], though conflicting results have been reported [21–23].

Palaeobotanical studies are promising because early fossils are filling gaps between these morphologically isolated families. Many gnetalean macrofossil plants have been reported from the Lower Cretaceous in Australia [24], Europe [25, 26], North America [25–28], South America [29–37], and particularly the Yixian Formation of western Liaoning, northeastern China [15, 38–54]. These fossil plants display a wide range of morphological diversity including leaf/bract and cone morphology, and are important in evolutionary studies of the early gnetophytes. Previously reported fossils are mainly from the Jianshangou Bed (ca. 125 Ma) of the Yixian Formation [55, 56], but none from younger strata such as the Jiufotang Formation (ca. 120.3 Ma) [57]. The evolution of the Jehol Biota consists of three phases, i.e. initiation during the Dabeigou phase, radiation in the Yixian phase, and decline in the Jiufotang phase [57]. Almost all known Cretaceous gnetalean fossils in northeastern China were found in the Yixian Formation. Any fossils from the Dabeigou phase and the Jiufotang phase would be interesting because they might provide some clues on the evolution and ecology of early gnetophytes.

Here we describe a new macrofossil plant from the Jiufotang Formation, and note that this species possessed certain unusual characters which distinguish it from all other known ephedroid species from the Jianshangou Bed.

## Results

### Gymnosperms

**Subclass** − Gnetidae

**Order** − Ephedrales

**Family** − Ephedraceae Dumortier

**Genus** − **Jianchangia** Y. Yang, Y.W. Wang & D.K. Ferguson, gen. nov.

**Diagnosis** − Fossil genus showing ephedroid morphology similar to *Gurvanella*, *Callianthus*, *Beipiaoa*, and *Ephedra* in having an articulated shoot with swollen nodes, longitudinally furrowed internodes, foliar organs having two parallel veins, and a compact ovulate cone having a pair of chlamydosperms, but distinguished by having linear bracts inserted in whorls.

**Etymology** − After the county name of the type locality, Jianchang.

**Fig. 1:**
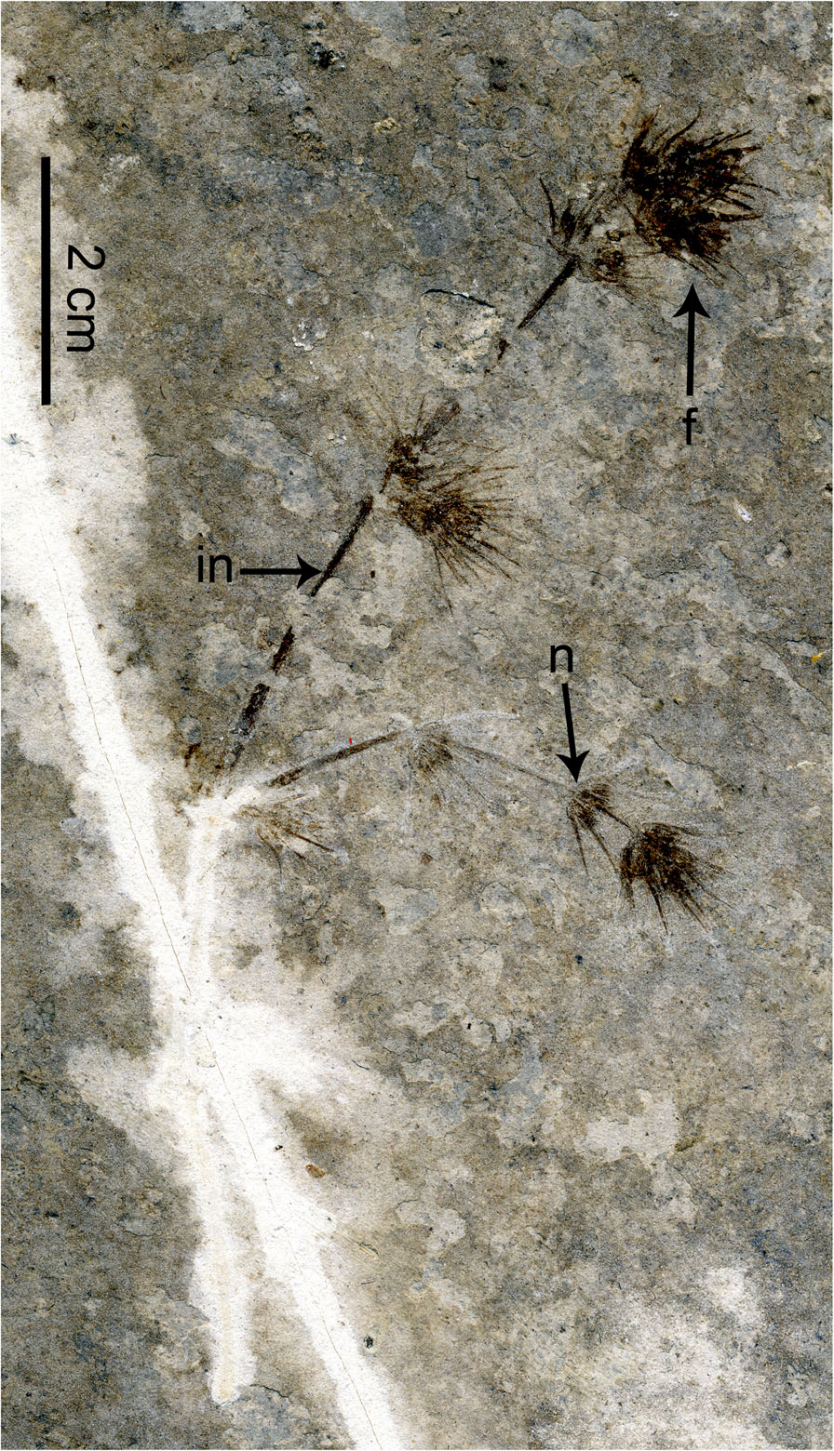
Type specimen of *Jianchangia verticillata* gen. et sp. nov. displaying the general morphology of the species. *Abbreviations*: in, internode; n, node; f, ovulate cone

**Fig. 2:**
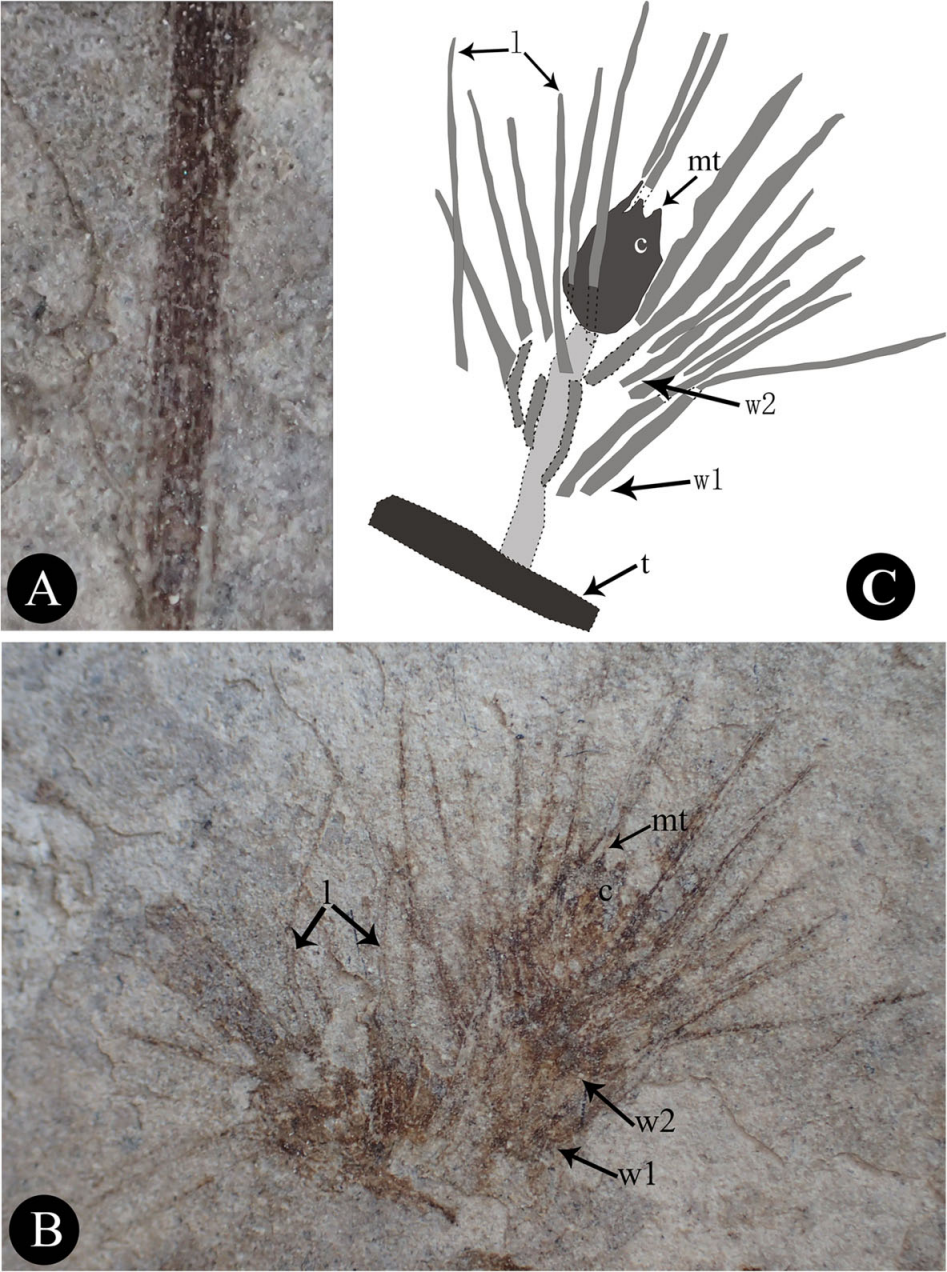
Morphological details of *Jianchangia verticillata* gen. et sp. nov. **a** a stem portion displaying the multiple longitudinal striations; **b** ovulate cones displaying the two whorls of linear bracts and the enclosed chlamydosperms; **c** diagram of an ovulate cone in (**b**) displaying structural details. *Abbreviations*: l, parallel-veined leaves/bracts; mt, micropylar tube; w1, the proximal whorl of bracts; w2, the distal whorl of bracts

**Fig. 3:**
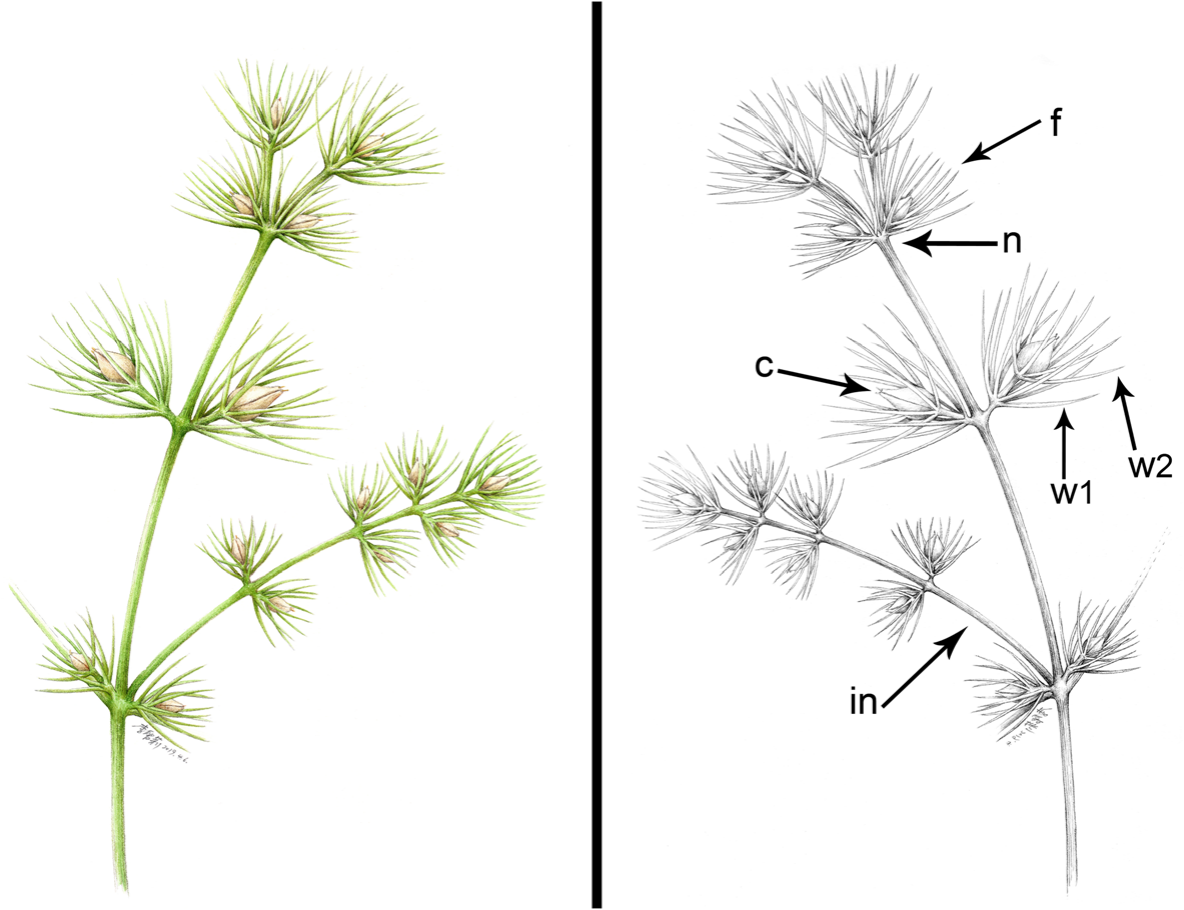
Reconstruction of *Jianchangia verticillata* gen. et sp. nov., displaying important characters. *Abbreviations*: c, chlamydosperms; f, ovulate cone; in, internode; n, node; w1, the proximal whorl of bracts; w2, the distal whorl of bracts

**Type Species** − **Jianchangia verticillata** Y. Yang, Y.W. Wang & D.K. Ferguson, sp. nov., Figs. 1, 2 and 3

**Diagnosis** – Ovulate cones sessile or subsessile; each possessing two whorls of bracts and two chlamydosperms; bracts leaf-like, linear-lanceolate, parallel-veined, ascending; chlamydosperms ca. 1.8 mm long, 1.5 mm wide.

**Description −** A single impression specimen with no counterpart showing a portion of a reproductive branch, ca. 9.1 cm long, ca. 1 mm thick but thinner distally, and the main branch with four nodes and one lateral branch. Internodes becoming shorter apically, basal internode ca. 3.9 cm long, next internode ca. 2.4 cm long, third internode ca. 2.1 cm long, fourth internode (or peduncle of an ovulate cone) ca. 0.6 cm long, and uppermost internode very short. Basal lateral branch ca. 3.2 cm long, and possessing four nodes, internodes becoming shorter distally, ca. 1.4 cm, 1.1 cm, and 0.5 cm long from the proximal to the distal, uppermost internode very short. Internodes possessing fine longitudinal striations (Figs. 1 and 2a). Ovulate cones sessile or sub-sessile and paired at each node (Figs. 1 and 2b). Each ovulate cone containing two whorls of bracts; four or more bracts at each node, linear, ascending. Two chlamydosperms enclosed within the uppermost whorl of bracts; obovoid, ca. 1.8 mm long, 1.5 mm wide, and narrowing towards the apex into a short tube (Fig. 2b).

**Distribution −** This species is only known from Lamadong Village, Jianchang County, Huludao Municipality, Liaoning of northeastern China.

**Holotype −** PE2018013101 (Fig. 1), deposited in the National Museum of Plant History of China, Institute of Botany, Chinese Academy of Sciences, Beijing.

**Etymology −** ‘*Verticillata*’ refers to the whorled linear bracts of the new species.

**Stratigraphic age and horizon −** Early Cretaceous (Aptian, ca. 120.3 Ma), Jiufotang Formation.

**Remarks −** Gnetophytes are common floristic elements in the Lower Cretaceous. They have been found in Europe, North America, South America, Australia, and northeastern Asia including Mongolia and northeastern China [25, 26, 30, 35, 38, 39, 41, 46, 47, 53]. The richest diversity is in the Yixian Formation [16]. Previous findings of ephedroid plants in northeastern China are restricted to Yixian and Lingyuan Counties, Liaoning Province. The new species described here was discovered in Jianchang County, Liaoning Province, China. This locality belongs to the Jiufotang Formation, which is younger than the Yixian Formation [55, 56].

**Fig. 4:**
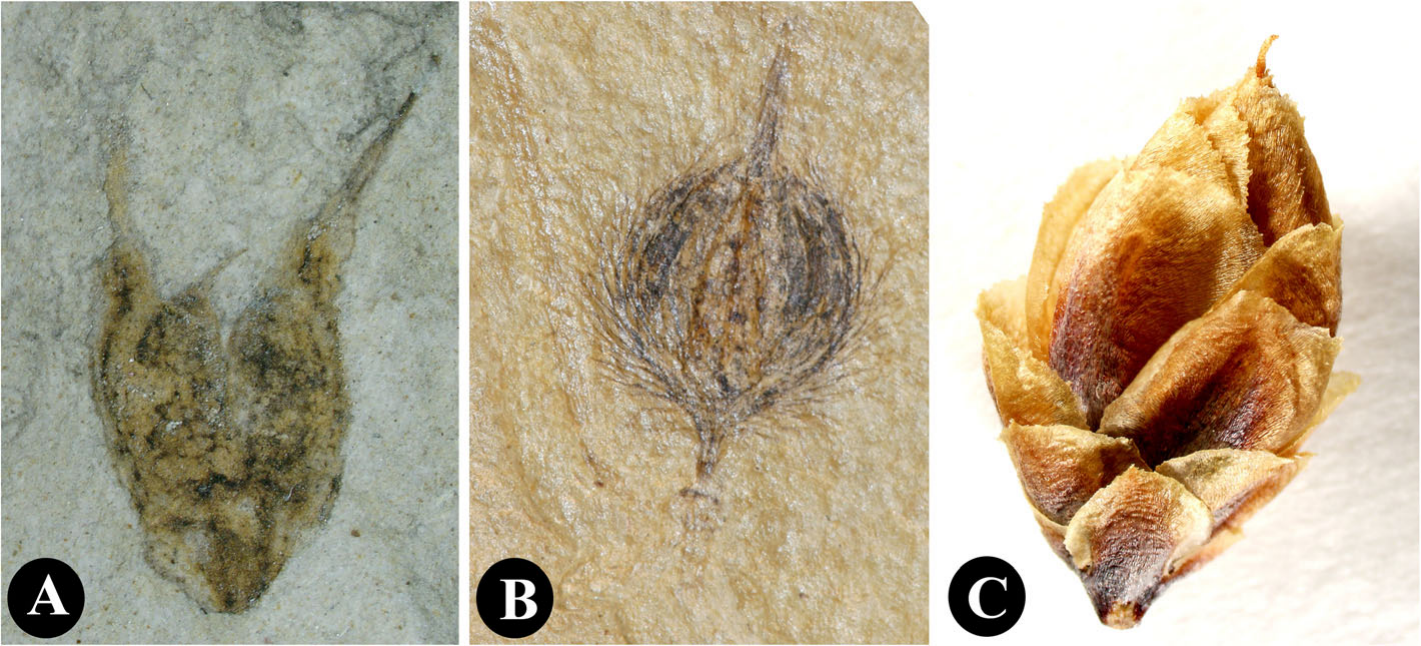
Ovulate cones displaying variation of bracts in genera related to *Jianchangia* gen. nov. **a** *Beipiaoa spinosa* Dilcher & al. displaying spinose bracts; **b** *Gurvanella dictyoptera* Krassilov displaying a bract having many furcate veins, **c** *Ephedra torreyana* S. Watson displaying obtuse bracts

The unique character of our new species lies in the number of foliar organs at the nodes. Though leaves/bracts are linear and possess two parallel veins as in many other gnetophyte species from the Yixian Formation, four or more of them are whorled at the nodes. The ovulate cones possess two whorls of bracts that are ascending and free from one another. There are two chlamydosperms enclosed in the uppermost whorl of bracts, which shows similarity to *Gurvanella*, *Callianthus*, *Beipiaoa*, and *Ephedra* [41, 46, 47, 51, 58]. These genera are classified based on their bract morphology (Figs. 4a−c). Our new species shows unusual bract morphology different from these known genera. As a result, we established a new genus here to include the new species based on the current classification of the ephedroid plants.

A one to one relationship between chlamydosperm and its subtending bract is relatively stable in ephedroid plants excepting *Siphonospermum*, in which chlamydosperms are pedicelled and have no associated bracts [45]. A chlamydosperm is axillary to a subtending bract and a pair of fertile bracts usually enclose two chlamydosperms in an ovulate cone. Sometimes, the paired bracts enclose only a single chlamydosperm because of abortion, e.g. *E. monosperma* and *E. nebrodensis*. The unusual feature of our new genus is that the multiple verticellate bracts enclose only two chlamydosperms in an ovulate cone, which fundamentally breaks the rule of chlamydosperm-bract relationship in Ephedraceae. There are two explanations for this pattern: the first is that the multiple bracts in a whorl are derived by division of two bracts, so there are only two chlamydosperms; the second is that the bracts are primary, but the original multiple chlamydosperms are reduced to only two due to loss. We are inclined to the first explanation, and believe that the multiple bracts of the same whorl may have been divided/multiplied from two bracts, since opposite phyllotaxis is predominant and presumably ancestral in the gnetophytes.

## Discussion

### Variation of leaves of early ephedroid plants

Ephedroid plants from the Yixian Formation display a great diversity of leaf morphology. Many of them possess linear and opposite leaves with parallel veins, e.g. *Liaoxia cheniae* (Wu & Guo) Rydin & al. [46], *Ephedra hongtaoi* Wang & Zheng [49], *Siphonospermum simplex* Rydin & Friis [45], *Chengia laxispicata* Y. Yang & al. [16], *Protognetella minuta* Krassilov [15], *Liaoningia decussata* Y. Yang & al. [59], but variation does occur. In *Spinobractea lanceolata* H.M. Liu & al. [43] and *Constrobilus ovata* H.M. Liu & al. [43], the leaves are broad and petiolate and have furcate-pinnate venation. In *Latibractea divisa* H.M. Liu & al. [43], the leaves are petiolate and divided and seem to be compound. In *Ephedra multinervia* Y. Yang & al., the leaves are strap-shaped and elongate and have multiple parallel veins connected by cross-veins, similar to those of Welwitschiaceae [52]. *Jianchangia verticillata* possesses normal ephedroid leaf shape, i.e. linear and parallel-veined, but is distinct from all known gnetalean fossils in that the foliar organs are verticellate.

### Morphological diversification of bracts of early ephedroid plants

**Fig. 5:**
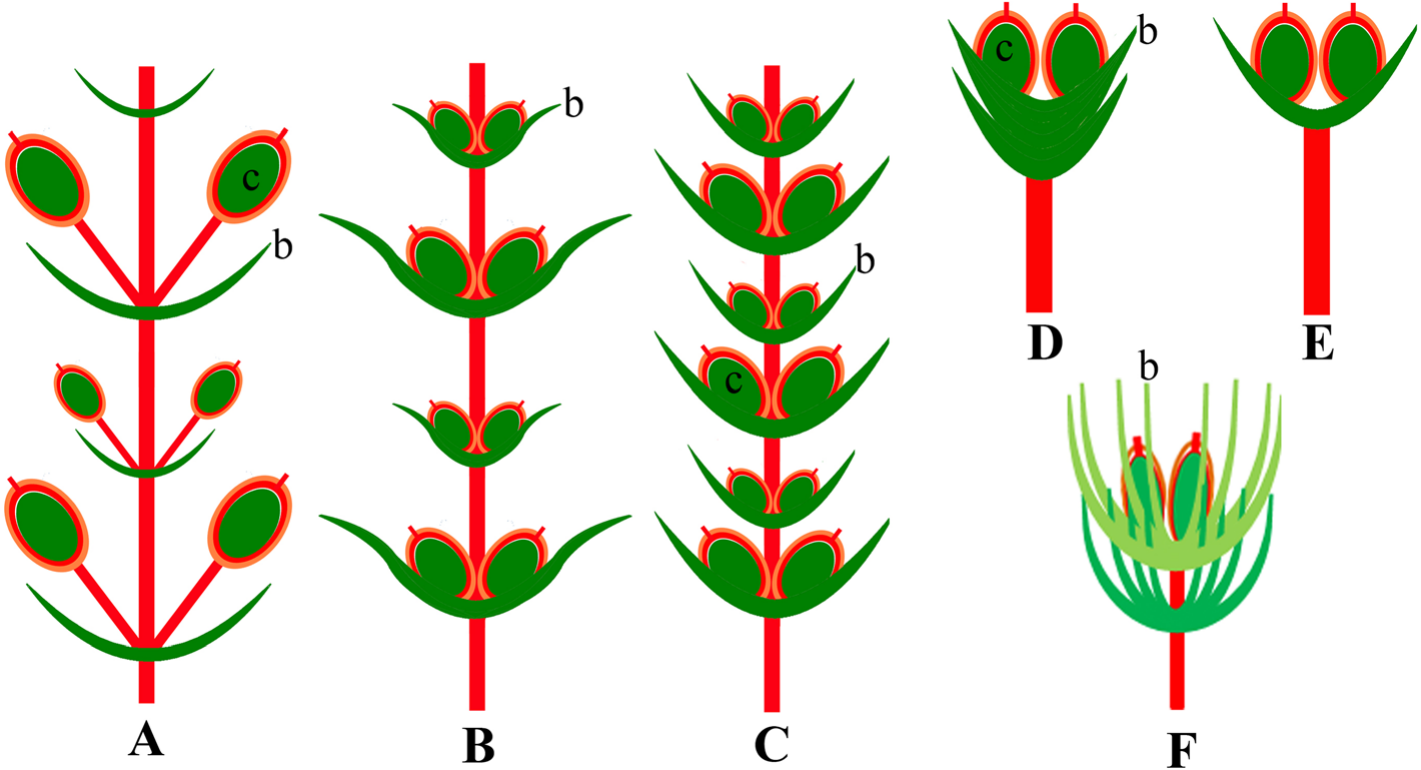
Illustrations of representative types of ovulate cones of ephedroid plants. A, *Siphonospermum*, reproductive shoot with pedicelled chlamydosperms; B, *Prognetella*, reproductive shoot with axillary and sessile chlamydosperms subtended by elongate leaf-like bracts; C, loosely arranged ovulate spike of *Chengia* and *Liaoningia* with shortened internodes and modified bracts; D, ovulate cone of *Ephedra* with a few pairs of proximal modified sterile bracts and a distal pair of modified fertile bracts, each modified fertile bract subtending an axillary and sessile chlamydosperm; E, ovulate cone of *Gurvanella* and *Beipiaoa* with one apical pair of modified fertile bracts, each subtending an axillary and sessile chlamydosperm; F, *Jiangchangia*, ovulate cone with two whorls of leaf-like bracts enclosing two sessile chlamydosperms

Representative types of ephedroid ovulate cones are illustrated in Fig. 5; a comparison between *Jianchangia* and related fossils from northeastern China is provided in Tables 1 and 2. *Jianchangia* shows similarities to other known early ephedroid plants from northeastern China in the linear and parallel-veined leaves and the articulate reproductive shoots with swollen nodes and fine longitudinal striations. *Siphonospermum simplex* differs from all known ephedroid plants [16] in having no typical ovulate cones or spikes, the chlamydosperms being pedicelled and lacking subtending bracts/leaves [45]. *Pseudoephedra* was reported as *incertae sedis* [44], but may be related to *Siphonospermum* in that it shows general ephedroid morphology, and the chlamydosperms are pedicelled and not closely associated with any foliar organs. *Jianchangia* differs from *Siphonospermum* and *Pseudoephedra* in the presence of ovulate cones and the whorled bracts subtending the inner paired chlamydosperms.

**Table 1:** A comparison of morphological characters between *Jianchangia* and other gnetalean macrofossils from northeastern China

Taxon	Locality	Formation	Leaves	Bract present	Bract whorls	Bract position	Bract shape	Literature
*Alloephedra*	Yanji Basin, Jilin Province, China	Dalazi Formation, Aptian-Albian, Lower Cretaceous	ovate-triangular	yes	2	paired	modified	Tao & Yang 2003 [48]
*Beipiaoa*	Huangbanjigou, Shangyuan, Beipiao, Liaoning, China	Yixian Formation, equivalent to the Barremian, Lower Cretaceous	-	yes	1	paired	modified, spinose or acute	Sun & al. 2001 [47]
*Chengia*	Dawangzhangzi, Lingyuan, Liaoning, China	Yixian Formation, equivalent to the Barremian, Lower Cretaceous	linear	yes	4-8	paired	modified	Yang & al. 2013 [16]
*Constrobilus*	Beipiao, Liaoning Province	Jianshangou Bed, Barremian, Lower Cretaceous	petiolate, broad ovate, pinnately veined	yes	1	paired	modified	Liu & al. 2013 [43]
*Ephedra archaeorhytidosperma*	Huangbanjigou, Shangyuan, Beipiao, Liaoning, China	Yixian Formation, equivalent to the Barremian, Lower Cretaceous	acute, triangular?	yes	1(-2?)	paired	modified, acute	Yang & al. 2005 [51]
*E. carnosa*	Huangbanjigou, Shangyuan, Beipiao, Liaoning, China	Yixian Formation, equivalent to the Barremian, Lower Cretaceous	-	yes	1	paired or ternately whorled	modified, fleshy	Yang & Wang 2013 [54]
*E. hongtaoi*	Dawangzhangzi, Lingyuan, Liaoning, China	Yixian Formation, equivalent to the Barremian, Lower Cretaceous	-	yes	1	paired	modified	Wang & Zheng
*E. multinervia*	Dawangzhangzi Village, Songzhangzi Town, Lingyuan City, Chaoyang District, Liaoning Province, China	Daxinfangzi Bed, Yixian Formation, Barremian, Lower Cretaceous	long, strap-shaped, having multiple parallel veins	yes	1	paired	modified	Yang & al. 2015 [52]
*Gurvanella*	Chaoyang, Liaoning, China; Mongolia	Yixian Formation, equivalent to the Barremian, Lower Cretaceous	linear, having 2(?) parallel veins	yes	1	ternately whorled	modified with profusely branched veins	Sun & al. 2001 [47]
*Jianchangia*	Lamadong Village, Jianchang County, Liaoning Province, northeastern China	Jiufotang Formation, Aptian, Lower Cretaceous	linear, parallel-veined	yes	2	verticillate	linear, leaf-like	This study
*Latibractea*	near Beipiao, Liaoning Province	Jianshangou Bed, Barremian, Lower Cretaceous	leaves petiolate, divided, parallel-veined	yes	3 or 4	paired	modified	Liu & al. 2013 [43]
*Liaoxia cheniae*	Shangyuan Village of Beipiao, Liaoning, China	Yixian Formation, equivalent to the Barremian, Lower Cretaceous	linear, parallel-veined	yes	2-6	paired	modified	Rydin & al. 2006 [26, 46]
*Prognetella*	Huangbanjigou, Shangyuan, Beipiao, Liaoning, China	Yixian Formation, equivalent to the Barremian, Lower Cretaceous	linear, having 2-4 parallel veins	yes, in reproductive shoots but not in typical cone	-	paired	leaf-like	Yang & Ferguson 2015 [15]
*Protognetum jurassicum*	Daohugou, Ningcheng County, Chifeng City, Inner Mongolia Autonomous Region, China	Yixian Formation, equivalent to the Barremian, Lower Cretaceous	linear	yes	2	paired	linear, leaf-like	Yang & al. 2017 [1, 59]
*Pseudoephedra*	Dawangzhangzi Village, Lingyuan City, Liaoning Province, China	Yixian Formation, Barremian–Aptian, Lower Cretaceous	linear	probably no	-	-	-	Liu & Wang 2016 [44]
*Siphonospermum simplex*	Jianshangou, Beipiao, Chaoyang City, west Liaoning, China	Yixian Formation, equivalent to the Barremian, Lower Cretaceous	linear, having 3 parallel veined	no	-	-	-	Rydin & Friis 2010 [45]
*Spinobractea*	near Beipiao, Liaoning Province	Jianshangou Bed, Barremian, Early Cretaceous	broadly lanceolate, forked venation	yes	2-4	paired	lanceolate	Liu & al. 2013 [43]

**Table 2:** A comparison of morphological characters between *Jianchangia* and other gnetalean macrofossils from northeastern China

Taxon	Female cone present	Chlamydosperms pedicled	Chlamydosperms in a cone	Chlamydosperms sculptured	Literature
*Alloephedra*	yes, compact	no	2	no	Tao & Yang 2003 [48]
*Beipiaoa*	yes, compact	no	2	no	Sun & al. 2001 [47]
*Chengia*	yes, but laxly arranged into spikes	no	Many in multiple whorls	no	Yang & al. 2013 [16]
*Constrobilus*	yes, compact	no	2	no	Liu & al. 2013 [43]
*Ephedra archaeorhytidosperma*	yes, compact	no	1-2	transversely wrinkled	Yang & al. 2005 [51]
*E. carnosa*	yes, compact	no	1-3	no	Yang & Wang 2013 [54]
*E. hongtaoi*	yes, compact	no	2	no	Wang & Zheng
*E. multinervia*	yes, compact	no	2	no	Yang & al. 2015 [52]
*Gurvanella*	yes, compact	no	usually 3	no	Sun & al. 2001 [47]
*Jianchangia*	yes, compact	no	2	no	This study
*Latibractea*	yes, compact	no	-	-	Liu & al. 2013 [43]
*Liaoxia cheniae*	yes, compact	no	many	no	Rydin & al. 2006 [26, 46]
*Prognetella*	no, in reproductive shoots	no	paired at nodes of reproductive shoots	no	Yang & Ferguson 2015 [15]
*Protognetum jurassicum*	laxly arranged spikes	no	4-6 whorled at nodes of a reproductive shoot	no	Yang & al. 2017 [1, 59]
*Pseudoephedra*	no	yes	not organized into a cone	no	Liu & Wang 2016 [44]
*Siphonospermum simplex*	no	yes	pedicled	no	Rydin & Friis 2010 [45]
*Spinobractea*	yes, more or less in lax spikes	no	1-2	no	Liu & al. 2013 [43]

*Protognetum* is a Jurassic macrofossil that also possesses ephedroid vegetative morphology and ovulate spikes; the bracts are linear and leaf-like, but at each node there are only two bracts subtending a whorl of chlamydosperms; these characters indicate its relationship to the Gnetaceae clade [1]. Our new fossil genus *Jianchangia* can be easily distinguished from *Protognetum* by the fact that the whorled bracts subtend only two chlamydosperms. *Spinobractea* has ovulate spikes with multiple pairs of bracts that appear to be elongate and strap-shaped [43], characters which are different from the whorled linear bracts enclosing only two chlamydosperms in *Jianchangia*. *Gnetum* possesses ovulate spikes with multiple whorls of chlamydosperms, with each whorl subtended by a collar of bracts, while *Welwitschia* has ovulate cones with multiple pairs of fertile bracts each subtending an axillary chlamydosperm. The circular and paired bracts in *Gnetum* and *Welwitschia* distinguish them from our new genus.

*Jianchangia* is also similar to a few other macrofossils from the Lower Cretaceous Yixian Formation in its ephedroid vegetative morphology and presence of bracts in ovulate spikes/cones, e.g. *Prognetella* Krassilov [15], *Chengia* Y. Yang & al. [16], *Liaoningia* Y. Yang & L.B. Lin [59], and *Liaoxia* Cao & Wu [46], but differs from all of them in having only one fertile whorl in each ovulate cone (vs. multiple fertile pairs/whorls in each ovulate cone in the latter five genera) in addition to fine, linear bract morphology. In *Prognetella*, the bracts are usually elongate and leaf-like, only modified at the basal portion to subtend the partially enclosed chlamydosperms [15], whereas the bracts of *Jianchangia* are not modified and are organized into whorls and not pairs. In *Liaoningia*, *Chengia*, and *Liaoxia*, the bracts are modified and not leaf-like, and paired at the nodes. *Jianchangia* differs from all of them in having unspecialized bracts arranged into whorls.

*Jianchangia* is similar to *Gurvanella* Krassilov [41], *Beipiaoa* Dilcher & al. [47], and *Ephedra* in having ovulate cones with only one fertile whorl in addition to the ephedroid vegetative morphology, but differs from the latter genera mainly in having unspecialized and verticillate bracts [35, 40, 47, 49, 51, 52, 54]. In *Gurvanella*, the two to three bracts are specialized in shape and possess unusual furcate venation [47], which makes it easily distinguished from *Jianchangia*. In *Beipiaoa* and *Ephedra*, the bracts are also modified but only two or three are inserted at each node [47]. This is also the case in *Erenia* Krassilov and *Callianthus* Wang & Zheng, in which the bracts are not leaf-like but specialized and encapsulate a pair of chlamydosperms [58]. *Constrobilus* and *Latibractea* probably belong to this complex because both of them have ovulate cones with one pair of chlamydosperms, as in modern *Ephedra*, but their leaves are unusual, divided in *Latibractea* and broad and pinnately veined in *Constrobilus* [43]. The differences between *Jianchangia* and these two genera are obvious, the bracts of the latter two genera are specialized and paired, thus differing from the leaf-like and whorled bracts in *Jianchangia*.

In summary, bracts of these ephedroid macrofossils show intergrading modifications from absence to presence, and from leaf-like to specialized shape. These transformations together with the reduction of the fertile whorls of the ovulate spike may reflect the evolutionary stages and probable relationships among them, but phylogenetic analysis is needed to test the direction of these transformations.

There are a few other reported gnetalean fossils, but unfortunately we cannot compare them with our new fossil genus because these plants are either pollen-producing, e.g. *Khitania* Guo & al. [38] and *Eamesia* Y. Yang & al. [53], or they are mesofossil seeds/chlamydosperms with no diagnostic characters of bracts and ovulate cones, e.g. *Ephedra drewriensis* Rydin & al. [26], *E. portugallica* Rydin & al. [26]., and *Bicatia* Friis & al. [25].

### Ecology

**Fig. 6:**
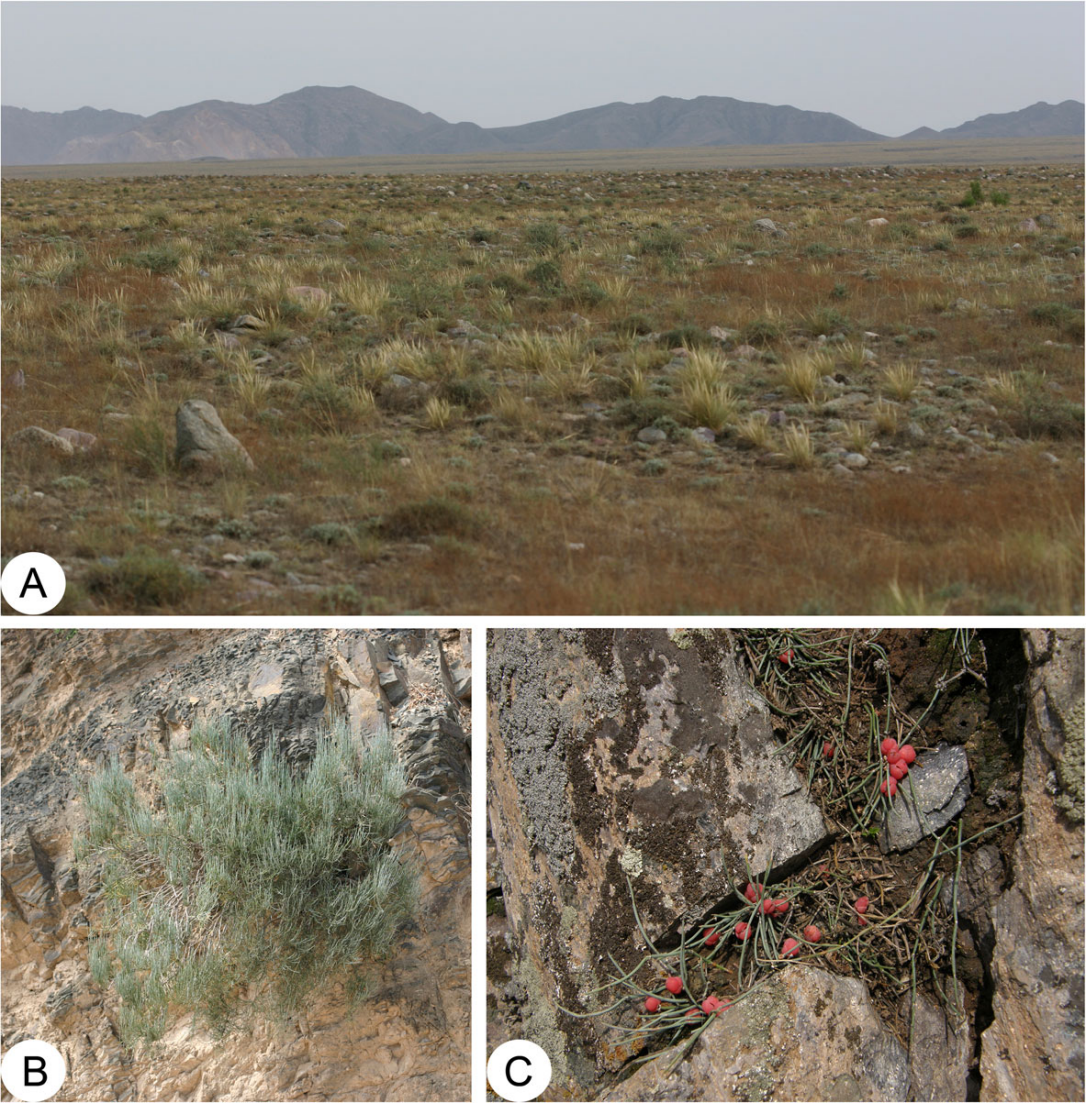
Habitats of representative species of living *Ephedra*. **a** *E. rhytidosperma* Pachom. in Gobi; **b** *E. equisetina* Bunge on cliffs; **c** *E. monosperma* Gmel. ex C.A. Mey. in stony crevices

Modern *Ephedra* usually lives in dry areas, in Gobi-type deserts (e.g. *E. rhytidosperma* Pachom., Fig. 6a), on cliffs (e.g. *E. equisetina* Bunge, Fig. 6b), or in stony crevices (e.g. *E. monosperma* Gmel. ex C.A. Mey., Fig. 6c), and they are distributed from Mediterranean regions eastwards to Siberia and northern China, in southwestern U.S.A. and northwestern Mexico, and Andean South America as well [60]. Early ephedroid plants may have occupied more diverse habitats than they do now, probably also in humid swampy places [15], and even in water [49], and these plants evolved a set of characters adapted to the palaeoenvironment. *Prognetella* is provided with an unusual set of characters, for instance, the fragmented reproductive shoots and frequent cystiform chlamydosperms may have facilitated the dispersal of diaspores in a lacustrine environment [15]. Our new species displays leaf/bract morphology distinct from other species of the Yixian Formation and extant species, which may represent an ecological adaptation. The numerous fine linear leaves of *Jianchangia* may be functionally analogous to the finely divided leaves of many living species of aquatic flowering plants, e.g. *Myriophyllum spicatum* L. and *Hippuris vulgaris* L. [61, 62].

Three phases of floristic development were recognized for the Jehol Biota, i.e. the early initiation Dabeigou phase, the middle radiation Yixian phase, and the late declining Jiufotang phase [57]. There are only a few plant fossils of gymnosperms recorded from the Jiufotang Formation, e.g. *Ginkgoites truncatus* Li, *Czekanowskia rigida* Heer, and *Elatocladus pinnatus* Sun & Zheng. All three plants were also recorded from the Jianshangou Bed, Yixian Formation, suggesting a floristic similarity between the Jiufotang Formation and the Yixian Formation. Though ephedroid macrofossils are common in the Yixian Formation, they have not been reported from the younger Jiufotang Formation thus far. Our new species is thus the first ephedroid species from the Jiufotang Formation. This finding confirms the floristic similarity and continuity between the Yixian Formation and the Jiufotang Formation.

The Yixian Formation displays an enormous diversity of ephedroid plants. This early burst of Ephedraceae may have been driven by the turbulent geological environment at the time [15]. It is well known that volcanic activity was frequent in the Yixian Formation [57]. This radiation was not only observed in the gnetophytes, but also in other plant groups, insects, mammals, dinosaurs, and birds [55]. In the younger Jiufotang Formation, the environment was relatively stable [57]. The declining species diversity in the Jiufotang Formation may be attributable to local extinction.

## Conclusions

Here we describe the first record of gnetophytes from the Lower Cretaceous Jiufotang Formation, Lamadong Village, Jianchang County, Liaoning Province, northeastern China. This finding demonstrates the lineage continuity of gnetophytes in western Liaoning since the Jiufotang Formation is ca. 5 Ma years younger than the Yixian Formation. This new plant *Jianchangia verticillata* gen. et sp. nov. also enhances our knowledge of morphological diversity in early gnetophytes. It has multiple foliar bracts, whorled at the nodes, and is distinguished from all other known ephedroid plants.

## Methods

**Fig. 7:**
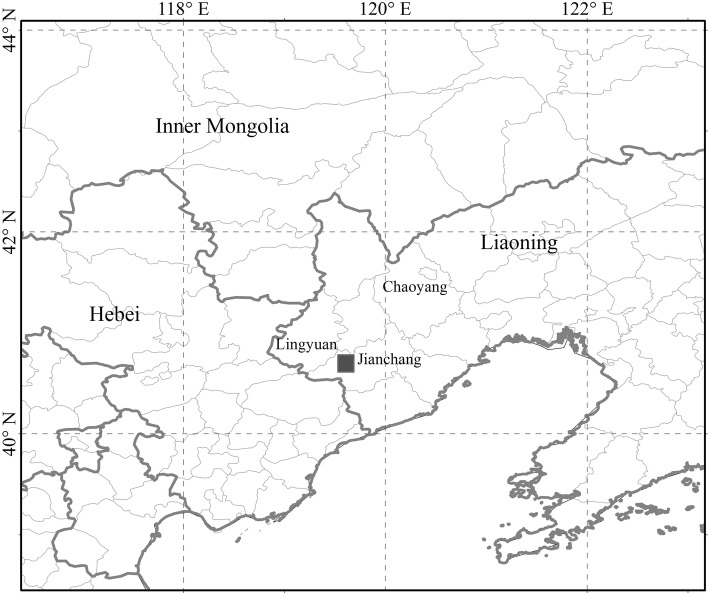
Type locality of *Jianchangia verticillata* gen. et sp. nov. The solid square (■) indicates the place where the species was collected (ESRI, Redlands, CA, USA; http://www.esri.com)

Our studied fossil was from Lamadong Village, Jianchang County, Liaoning Province, northeastern China (Fig. 7). The fossil bed belongs to the Jiufotang Formation. The Formation dates back to the Aptian, ca. 120.3 Ma [63], and is well-known for early birds, e.g. *Jianchangornis microdonta* Zhou & al. [64], *Bohaiornis guoi* Hu & al. [65], *Schirooura lii* Zhou & al. [66], *Jeholornis palmapenis* O’Connor & al. [67], *Zhongornis haoae* Gao & al. [68, 69], and the dinosaur *Moganopterus zhuiana* Lu & al. [70]. Plant fossils were rarely reported, and only a few gymnosperms have been recorded, e.g. *Ginkgoites truncatus* Li, *Czekanowskia rigida* Heer, and *Elatocladus pinnatus* Sun & Zheng [57]. The new fossil is deposited in the National Museum of Plant History of China, Institute of Botany, Chinese Academy of Sciences, Beijing, China. The plant fossil was preserved as impressions, and is only fragmentary.

The fossil was observed with a light microscope (Nikon Eclipse E600) and was photographed with digital cameras (Nikon D700 and Olympus TG-3). The figures were manually edited and created using Adobe Photoshop CS2 ver. 9.0 and CorelDRAW X4. The map of the type locality was generated using ArcGIS 9.3 (ESRI, Redlands, CA, USA; http://www.esri.com).

## Data Availability

All data and materials used in this study are included in this published article.

## Abbreviations

c: chlamydosperms
f: ovulate cone
in: internode
l: parallel-veined leaves/bracts
mt: micropylar tube
n: node
w1: proximal whorl of bracts
w2: distal whorl of bracts
